# On the Impossibility of Learning the Missing Mass

**DOI:** 10.3390/e21010028

**Published:** 2019-01-02

**Authors:** Elchanan Mossel, Mesrob I. Ohannessian

**Affiliations:** 1 Department of Mathematics, Massachusetts Institute of Technology, Cambridge, MA 02142, USA; 2 Toyota Technological Institute at Chicago, Chicago, IL 60637, USA

**Keywords:** missing mass, rare events, Good–Turing, light tails, heavy tails, no free lunch

## Abstract

This paper shows that one cannot learn the probability of rare events without imposing further structural assumptions. The event of interest is that of obtaining an outcome outside the coverage of an i.i.d. sample from a discrete distribution. The probability of this event is referred to as the “missing mass”. The impossibility result can then be stated as: the missing mass is not distribution-free learnable in relative error. The proof is semi-constructive and relies on a coupling argument using a dithered geometric distribution. Via a reduction, this impossibility also extends to both discrete and continuous tail estimation. These results formalize the folklore that in order to predict rare events without restrictive modeling, one necessarily needs distributions with “heavy tails”.

## 1. Introduction

Given data consisting of *n* i.i.d. observations $X_{1},\cdots ,X_{n}$ from an unknown distribution *p* over the positive integers $N_{+}$, we traditionally compute the *empirical distribution*:

$${\hat{p}}_{n}\left(x\right):=\frac{1}{n}\sum_{i=1}^{n}1\left\{X_{i}=x\right\}.$$

To estimate the probability $p\left(E\right):=\sum_{x\in E}p\left(x\right)$ of an event $E\subset N_{+}$, we could use ${\hat{p}}_{n}\left(E\right):=\sum_{x\in E}{\hat{p}}_{n}\left(x\right)$. This works well for abundantly represented events, but not as well for rare events. An unequivocally rare event is the set of symbols that are *missing* in the data,

$$E_{n}:=\left\{x\in N_{+}:\hat{p}\left(x\right)=0\right\}.$$

The probability of this (random) event is denoted by the *missing mass*:

$$M_{n}\left(X_{1},\cdots ,X_{n}\right):=p\left(E_{n}\right)=\sum_{x\in N_{+}}p\left(x\right)1\left\{\hat{p}\left(x\right)=0\right\}.$$

The question we strive to answer in this paper is: “Can we learn the missing mass when *p* is an arbitrary distribution on $N_{+}$?” Definition 1 phrases this precisely in the learning framework.

**Definition** **1.** *An* estimator *is a sequence of functions ${\hat{M}}_{n}\left(x_{1},\cdots ,x_{n}\right):N_{+}^{n}\to \left[0,1\right]$. We say that an estimator* learns *the missing mass in relative error with respect to a family P of distributions, if for every p∈P and every ϵ,δ>0 there exists $n_{0}\left(p,\epsilon ,\delta \right)$ such that for all $n>n_{0}\left(p,\epsilon ,\delta \right)$:*

$$P_{p}\left\{\left|\frac{{\hat{M}}_{n}\left(X_{1},\cdots ,X_{n}\right)}{M_{n}\left(X_{1},\cdots ,X_{n}\right)}-1\right|<\epsilon \right\}>1-\delta .$$

*The learning is said to be* distribution-free, *if P consists of* all *distributions on $N_{+}$.*

In this framework, our question becomes whether we can distribution-free learn the missing mass in relative error. It is obvious that the empirical estimator $\hat{p}\left(E_{n}\right)$ gives us the trivial answer of 0, and cannot learn the missing mass. A popular alternative is the Good–Turing estimator of the missing mass, which is the fraction of singletons in the data:

$$G_{n}:=\sum_{x\in N_{+}}\frac{1}{n}1\left\{n\hat{p}\left(x\right)=1\right\}.$$

The Good–Turing estimator has many interpretations. Its original derivation by Good [1] uses an empirical-Bayes perspective. It can also be thought of as a leave-one-out cross-validation estimator, which contributes to the missing set if and only if the holdout appears exactly once in the data. Fundamentally, $G_{n}$ derives its form and its various properties from the simple fact that:

$$E\left[G_{n}\right]=\sum_{x\in N_{+}}p\left(x\right){\left(1-p\left(x\right)\right)}^{n-1}=E\left[M_{n-1}\right].$$

A study of $G_{n}$ in the learning framework was first undertaken by McAllester and Schapire [2] and continued later by McAllester and Ortiz [3]. Some further refinement and insight was also given later by Berend and Kontorovich [4]. These works focused on additive error. Ohannessian and Dahleh [5] shifted the attention to relative error, establishing the learning property of the Good–Turing estimator with respect to the family of heavy-tailed (roughly power-law) distributions, e.g., $p\left(x\right)\propto x^{-1/\alpha }$ with α∈(0,1). This work also showed that Good–Turing *fails* to learn the missing mass for geometric distributions, and therefore does not achieve distribution-free learning. More recently, Ben-Hamou et al. [6] provide a comprehensive and tight set of concentration inequalities, which can be interpreted in the current framework, and which further demonstrate that Good–Turing can learn with respect to heavier-than-geometric light tails, e.g., the family that includes $p\left(x\right)\propto 2^{-x^{\gamma }}$ with γ∈(0,1) (see the definition in Section 4.3 and Remark 4.3 in that paper), in addition to power-laws.

These results leave open the important question: does there exist some *other* estimator that can learn the missing mass in relative error for *any* distribution *p*? Our contributions are:

- We prove that there are no such estimators, thus providing the first such “no free lunch” theorem for learning about rare events. The first insight to glean from this impossibility result is that one is justified to use further structural assumptions. Furthermore, the proof relies on an implicit construction that uses a dithered geometric distribution. In doing so, it shows that the failure of the Good–Turing estimator for light-tailed distributions is not a weakness of the procedure, but is rather due to a fundamental barrier. Conversely, the success of Good–Turing for heavier-than-geometric and power laws shows its universality, in some restricted sense. In particular, in concrete support to folklore (e.g., [7]), we can state that for estimating probabilities of rare events, heavy tails are both necessary and sufficient.
- We extend this result to continuous tail estimation.
- We show, on a positive note, that upon restricting to parametric light-tailed families learning may be possible. In particular, we show that for the geometric family the natural plug-in estimator learns the missing mass in relative error. As an ancillary result, we prove an instance-by-instance convergence rate, which can be interpreted as a weak sample complexity. For this, we establish some sharp concentration results for the gaps in geometric distributions, which may be of independent interest.

The paper is organized as follows. In Section 2, we present our main result, with a detailed exposition of the proof. In Section 3 we discuss questions of weak versus strong learnability, we give an immediate extension to continuous tail estimation, show that parametric light-tailed learning is possible, comment further on the Good–Turing estimator, and concisely place this result in the context of a chief motivating application, that of computational linguistics. Lastly, we conclude in Section 4 with a summary and open questions.

## 2. Main Result

Our main result is stated as follows. The rest of this section is dedicated to its detailed proof.

**Theorem** **1.** *There exists a positive ϵ>0 and a strictly increasing sequence ${\left(n_{k}\right)}_{k=1,2,\cdots }$, such that for every estimator ${\hat{M}}_{n}$ there exists a distribution $p^{⋆}$, such that for all k:*

(1) $$P_{p^{⋆}}\left\{\left|\frac{{\hat{M}}_{n_{k}}}{M_{n_{k}}}-1\right|>\epsilon \right\}>\epsilon .$$

*In particular, it follows that it is impossible to perform distribution-free learning of the missing mass in relative error.*

**Remark** **1.** *Our proof below implies the statement of the theorem with $\epsilon =10^{-4}$ and $n_{k}=6.5\times 2^{k}$, but we did not make an honest effort to optimize these parameters.*

### 2.1. Proof Outline

Consider the family $P_{\beta ,m}$ of β,m-dithered geometric$\left(\frac{1}{2}\right)$ distributions, where the mass of each outcome beyond a symbol *m* of a $\mathrm{geometric}(\frac{1}{2})$ random variable is divided between two symbols, with a fraction β in one and 1−β in the other. The individual distributions in this family differ only by which of each pair of such symbols gets which fraction. More precisely:

**Definition** **2.** *The β,m-dithered geometric$\left(\frac{1}{2}\right)$ family, for a given choice of $\beta \in (0,\frac{1}{2})$ and $m\in N_{+}$, is a collection of distributions parametrized by the dithering choices $\theta \in {\left\{\beta ,1-\beta \right\}}^{N_{+}}$, $\theta :=(\theta_{1},\theta_{2},\cdots ,\theta_{j},\cdots )$, as follows:*

(2) $$\begin{array}{c} P_{\beta ,m}=\left\{p_{\theta }:p_{\theta }\left(x\right)=\frac{1}{2^{x}},x=1,\cdots ,m;\right.\\ \;\left.p_{\theta }\left(m+2j-1\right)=\frac{\theta_{j}}{2^{m+j}},\;p_{\theta }\left(m+2j\right)=\frac{1-\theta_{j}}{2^{m+j}},\;j\in N_{+},\;\theta \in {\left\{\beta ,1-\beta \right\}}^{N_{+}}\right\}. \end{array}$$

The intuition of the proof of Theorem 1 is that within such light-tailed families, two distributions may have very similar samples and thus estimated values, yet have significantly different true values of the missing mass. This follows the general methodology of many statistical lower bounds. We now state the outline of the proof. We choose a subsequence of the form $n_{k}=C2^{k}$. We set β=1/4, m=1, and C=6.5. The value of ϵ>0 is made explicit in the proof, and depends only on these choices. We proceed by induction.

- We show that there exists $\theta_{1}^{⋆}$ such that for all θ with $\theta_{1}=\theta_{1}^{⋆}$ we have for $n=n_{1}$:
  (3) $$P_{p_{\theta }}\left\{\left|\frac{{\hat{M}}_{n}}{M_{n}}-1\right|>\epsilon \right\}>\epsilon .$$
- Then, at every step k>1:
  (H) We start with $\left(\theta_{1}^{⋆},\cdots ,\theta_{k-1}^{⋆}\right)$ such that for all θ with $(\theta_{1},\cdots ,\theta_{k-1})=$$\left(\theta_{1}^{⋆},\cdots ,\theta_{k-1}^{⋆}\right)$, Inequality (3) holds for $n=n_{1},\cdots ,n_{k-1}$.
  (*) We then show that it must be that for at least one of $\tilde{\theta }=\beta$ or $\tilde{\theta }=1-\beta$, for all θ with $\left(\theta_{1},\cdots ,\theta_{k}\right)=\left(\theta_{1}^{⋆},\cdots ,\theta_{k-1}^{⋆},\tilde{\theta }\right)$, Inequality (3) holds additionally for $n=n_{k}$. We select $\theta_{k}^{⋆}$ to be the corresponding $\tilde{\theta }$.
- This induction produces an infinite sequence $\theta^{⋆}\in {\left\{\beta ,1-\beta \right\}}^{N_{+}}$, and the desired distribution in Theorem 1 can be chosen as $p^{⋆}=p_{\theta^{⋆}}$, since it is readily seen to satisfy the claim for each $n_{k}$, by construction.

### 2.2. Proof Details

We skip the proof of the base case, since it is mostly identical to that of the induction step. Therefore, in what follows we assume that $\left(\theta_{1}^{⋆},\cdots ,\theta_{k-1}^{⋆}\right)$ satisfies the inductive hypothesis (H), and we would like to prove that the selection in (*) can always be done. Let us denote the two choices of parameters by

$$\theta :=(\theta_{1}^{⋆},\cdots ,\theta_{k-1}^{⋆},\beta ,\theta_{k+1},\cdots ),$$

and

$$\theta^{\prime }:=\left(\theta_{1}^{⋆},\cdots ,\theta_{k-1}^{⋆},1-\beta ,\theta_{k+1}^{\prime },\cdots \right),$$

and let us refer to $\left(\theta_{k+1},\cdots \right)$ and $\left(\theta_{k+1}^{\prime },\cdots \right)$ by the *trailing parameters*. What we show in the remainder of the proof is that with two arbitrary sets of trailing parameters, we cannot have two simultaneous violations of Inequality (3) (for both θ and $\theta^{\prime }$). That is, we cannot have both:

(4) $$P_{p_{\theta }}\left\{\left|\frac{{\hat{M}}_{n_{k}}}{M_{n_{k}}}-1\right|>\epsilon \right\}<\epsilon \;\mathrm{and}\;P_{p_{\theta^{\prime }}}\left\{\left|\frac{{\hat{M}}_{n_{k}}}{M_{n_{k}}}-1\right|>\epsilon \right\}<\epsilon .$$

This is stated in Lemma 3, in the last portion of this section. To see why this is sufficient to show that the selection in (*) can be done, consider first the case that Inequality (3) with $n=n_{k}$ is upheld for both θ and $\theta^{\prime }$ with any two sets of trailing parameters. In this case we can arbitrarily choose $\theta_{k}^{⋆}$ to be either β or 1−β, since the induction step is satisfied. We can therefore focus on the case in which this fails. That is, for either θ or $\theta^{\prime }$ a choice of trailing parameters can be made such that Inequality (3) with $n=n_{k}$ is *not* satisfied, and therefore one of the two cases in (4) holds [say, for example, for θ]. Fix the corresponding trailing parameters [in this example, $\left(\theta_{k+1},\cdots \right)$]. Then, for *any* choice of the *other* set of trailing parameters [in this example, $\left(\theta_{k+1}^{\prime },\cdots \right)$], Lemma 3 precludes a violation of Inequality (3) for $n=n_{k}$ by the other choice [in this example, $\theta^{\prime }$]. Therefore this choice can be selected for $\theta_{k}$ [in this example, $\theta_{k}=1-\beta$.]

By using the *coupling* device and restricting ourselves to a *pivotal event*, we formalize the aforementioned intuition that the estimator may not distinguish between two separated missing mass values, and deduce that both statements in (4) cannot hold simultaneously.

#### 2.2.1. Coupling

**Definition** **3.** *A* coupling *between two distributions p and $p^{\prime }$ on $N_{+}$ is a joint distribution q on $N_{+}^{2}$, such that the first and second marginal distributions of q revert back to p and $p^{\prime }$ respectively.*

Couplings are useful because probabilities of events on each side may be evaluated on the joint probability space, while forcing events of interest to occur in an orchestrated fashion. Going back to our induction step and the specific choices θ and $\theta^{\prime }$ with arbitrary trailing parameters, we perform the following coupling. For $\left(x,x^{\prime }\right)\in N_{+}^{2}$, let

(5) $$q\left(x,x^{\prime }\right)=\left\{\begin{array}{ccc} p_{\theta }\left(x\right)=p_{\theta^{\prime }}\left(x^{\prime }\right) & ; & \mathrm{if}x=x^{\prime }<m+2k-1;\\ \beta /2^{m+k} & ; & \mathrm{if}x=x^{\prime }=m+2k-1,\mathrm{or}\mathrm{if}x=x^{\prime }=m+2k,\\ \left(1-2\beta \right)/2^{m+k} & ; & \mathrm{if}x=m+2k\mathrm{and}x^{\prime }=m+2k-1;\\ p_{\theta }\left(x\right)p_{\theta^{\prime }}\left(x^{\prime }\right)/2^{m+k} & ; & \mathrm{if}x,x^{\prime }>m+2k;\\ 0 & ; & \mathrm{otherwise}. \end{array}\right.$$

It is easy to verify that *q* in Equation (5) is a coupling between $p_{\theta }$ and $p_{\theta^{\prime }}$ as in Definition 3. Now let us observe the consequences of this choice. If $X,X^{\prime }$ are generated according to *q*, then if either is in {1,⋯,m+2k−2} then both values are *identical*. If either is in {m+2k+1,⋯} then so is the other, but otherwise the two values are conditionally independent. If either is in {m+2k−1,m+2k}, so is the other, and the conditional probability is given by:

$x^{\prime }$m+2k−1m+2k*x*
m+2k−1β0m+2k1−2ββ

Now consider coupled data ${\left(X_{i},X_{i}^{\prime }\right)}_{i=1,\cdots ,n}$ generated as i.i.d. samples from *q*. It follows that, marginally, the *X*-sequence is i.i.d. from $p_{\theta }$, and so is the $X^{\prime }$-sequence from $p_{\theta^{\prime }}$. Any event *B* that is exclusively *X*-measurable or $B^{\prime }$ that is exclusively $X^{\prime }$-measurable has the same probability under the coupled measure. That is,

$$P_{p_{\theta }}\left(B\right)=P_{q}\left(B\right):=q^{n}\left(B\times N_{+}^{n}\right)$$

and

$$P_{p_{\theta^{\prime }}}\left(B^{\prime }\right)=P_{q}\left(B^{\prime }\right):=q^{n}\left(N_{+}^{n}\times B^{\prime }\right).$$

In what follows we work only with coupled data, and use simply the shorthand P to mean $P_{q}$.

#### 2.2.2. Pivotal Event

The event we would like to work under is that of the coupled samples being identical, while exactly covering the range 1, ⋯, m+2k−1. Let’s call this the *pivotal event* and denote it by:

(6) $$A_{k}:=\overset{n_{k}}{\underset{i=1}{⋂}}\left\{X_{i}=X_{i}^{\prime }\right\}\;\;\cap \;\;\left\{\left\{X_{1},\ldots ,X_{n_{k}}\right\}=\left\{1,\cdots ,m+2k-1\right\}\right\}.$$

The reason $A_{k}$ interests us is that it encapsulates the aforementioned intuition.

**Lemma** **1.** *Under event $A_{k}$, the coupled missing masses are distinctly separated,*

$$\frac{M_{n_{k}}}{M_{n_{k}}^{\prime }}=\frac{2-\beta }{1+\beta },$$

*while any estimator cannot distinguish the coupled samples,*

$${\hat{M}}_{n_{k}}={\hat{M}}_{n_{k}}^{\prime }.$$

**Proof.**  The confusion of any estimator is simply due to the fact that under $A_{k}$, the coupling forces all samples to be identical $X_{i}=X_{i}^{\prime }$, for all $i=1,\cdots ,n_{k}$.

Thus ${\hat{M}}_{n_{k}}={\hat{M}}_{n_{k}}^{\prime }$, since estimators only depend on the samples and not the probabilities.

The missing masses, on the other hand, do depend on both the samples and the probabilities and thus they differ. But the event $A_{k}$ makes the set of missing symbols simply the tail m+2k,m+2k+1,⋯, so we can compute the missing masses exactly:

$$M_{n_{k}}=p_{\theta }\left(m+2k\right)+\sum_{x=m+2k+1}^{\infty }p_{\theta }\left(x\right)=\frac{1-\theta_{k}}{2^{m+k}}+\frac{1}{2^{m+k}}=\left(2-\beta \right)2^{-m-k},\mathrm{and}$$

$$M_{n_{k}}^{\prime }=p_{\theta^{\prime }}\left(m+2k\right)+\sum_{x=m+2k+1}^{\infty }p_{\theta^{\prime }}\left(x\right)=\frac{1-\theta_{k}^{\prime }}{2^{m+k}}+\frac{1}{2^{m+k}}=\left(1+\beta \right)2^{-m-k},$$

where $\frac{1}{2^{m+k}}$ follows from the usual geometric sum. The claim follows. □

We now show that $A_{k}$ has always a positive probability, bounded away from zero.

**Lemma** **2.** *For β=1/4, m=1, C=6.5 and $n_{k}=C2^{k}$, there exists a positive constant η>0 that does not depend on θ, such that for all k, $P(A_{k})>\eta$. We can explicitly set $\eta =2\cdot 10^{-4}$.*

**Proof.**  Please note that $A_{k}$ in Equation (6) overspecifies the event. In fact, only forcing the exact coverage of 1,⋯,m+2k−1 is sufficient, since this implies in turn that the coupled samples are identical. Recalling the coupling of Equation (5), this is immediate for symbols in 1,⋯,m+2k−2, and follows for m+2k−1 since m+2k is not allowed in this event. We can then write $A_{k}=A_{k,1}\cap A_{k,2}$, dividing the exact coverage to the localization in the range and the representation of each symbol by at least one sample:

$$\begin{array}{cc} A_{k,1}=\left\{{⋃}_{i=1}^{n_{k}}\left\{X_{i}\right\}\subseteq \left\{1,\cdots ,m+2k-1\right\}\right\} & \;(\mathrm{localization}),\\ A_{k,2}=\left\{{⋃}_{i=1}^{n_{k}}\left\{X_{i}\right\}⊇\left\{1,\cdots ,m+2k-1\right\}\right\} & \;(\mathrm{representation}). \end{array}$$

Let α be the probability of $\left(X_{i},X_{i}^{\prime }\right)$ for a given *i* being in {(1,1),⋯,(m+2k−1,m+2k−1)}. From the coupling in Equation (5) and the structure of the dithered family in Equation (2), we see that for up to m+2k−2 this probability sums up to the m+k−1 first terms of a geometric$\left(\frac{1}{2}\right)$, and for (m+2k−1,m+2k−1) the coupling assigns it $\beta /2^{m+k}$, thus:

$$\alpha =\sum_{x=1}^{m+2k-1}q\left(x,x\right)=1-\frac{1}{2^{m+k-1}}+\frac{\beta }{2^{m+k}}.$$

We can then explicitly compute:

$$P\left(A_{k,1}\right)=\alpha^{n_{k}}={\left(1-\frac{1}{2^{m+k-1}}+\frac{\beta }{2^{m+k}}\right)}^{n_{k}}=:\eta_{1}\left(k\right).$$

Meanwhile, the complement of $A_{k,2}$ is the event that at least one of {(1,1),⋯,(m+2k−1,m+2k−1)} does not appear, that is $A_{k,2}^{c}={⋃}_{x=1}^{m+2k-1}\left\{x\notin \cup \{X_{i}\}\right\}$. Conditionally on $A_{k,1}$, the occurrence probabilities of these symbols are simply normalized by α, that is $P\left(x\notin \cup X_{i}|A_{k,1}\right)={\left[1-q(x,x)/\alpha \right]}^{n_{k}}$. Thus, by a union bound, we have:

$$\begin{array}{ccc} P(A_{k,2}|A_{k,1}) & = & 1-P(A_{k,2}^{c}|A_{k,1})\\ & \geq & 1-\sum_{x=1}^{m+2k-1}P\left(x\notin \cup X_{i}|A_{k,1}\right)\\ & = & 1-\sum_{x=1}^{m+2k-1}{\left[1-q(x,x)/\alpha \right]}^{n_{k}}\\ & \geq & 1-\sum_{x=1}^{m+2k-1}{\left[1-q(x,x)\right]}^{n_{k}}\\ & = & 1-\sum_{x=1}^{m}{\left(1-\frac{1}{2^{x}}\right)}^{n_{k}}\\ & & -\sum_{j=1}^{k-1}\left[{\left(1-\frac{\beta }{2^{m+j}}\right)}^{n_{k}}+{\left(1-\frac{1-\beta }{2^{m+j}}\right)}^{n_{k}}\right]-{\left(1-\frac{\beta }{2^{m+k}}\right)}^{n_{k}}\\ & \geq & 1-\sum_{x=1}^{m}{\left(1-\frac{1}{2^{x}}\right)}^{n_{k}}-2\sum_{j=1}^{k-1}{\left(1-\frac{\beta }{2^{m+j}}\right)}^{n_{k}}-{\left(1-\frac{\beta }{2^{m+k}}\right)}^{n_{k}}=:\eta_{2}\left(k\right), \end{array}$$

where the last inequality follows from the fact that $\beta <\frac{1}{2}$. Therefore,

$$P\left(A_{k}\right)=P\left(A_{k,1}\cap A_{k,2}\right)=P\left(A_{k,1}\right)P\left(A_{k,2}|A_{k,1}\right)\geq \eta_{1}\left(k\right)\eta_{2}\left(k\right)\geq \underset{k\geq 1}{\inf}\eta_{1}\left(k\right)\eta_{2}\left(k\right)=:\eta .$$

We now use our choices of β=1/4, m=1, C=6.5, and $n_{k}=C2^{k}$, to bound this worst-case η. In particular, we can verify that $\eta \geq 2\cdot 10^{-4}$, and it follows as claimed that the pivotal event has always a probability bounded away from zero. □

#### 2.2.3. Induction Step

We now combine all the elements presented thus far to complete the proof of Theorem 1 by establishing the following claim, which we have shown in the beginning of the detailed proof section to be sufficient for the validity of the induction step. In particular, we restate Equation (4) under the coupling of Equation (5).

**Lemma** **3.** *Let*

$$\theta :=\left(\theta_{1}^{⋆},\cdots ,\theta_{k-1}^{⋆},\beta ,\theta_{k+1},\cdots \right),\;\mathrm{and}\;\theta^{\prime }:=\left(\theta_{1}^{⋆},\cdots ,\theta_{k-1}^{⋆},1-\beta ,\theta_{k+1}^{\prime },\cdots \right),$$

*with* arbitrary *trailing parameters $\left(\theta_{k+1},\cdots \right)$ and $\left(\theta_{k+1}^{\prime },\cdots \right)$. Let q be the coupling of Equation (5), and let $B_{k}=\left\{\left|{\hat{M}}_{n_{k}}/M_{n_{k}}-1\right|>\epsilon \right\}$ and $B_{k}^{\prime }=\left\{\left|{\hat{M}}_{n_{k}}^{\prime }/M_{n_{k}}^{\prime }-1\right|>\epsilon \right\}$. Then given our choices of β=1/4, m=1, C=6.5 and $n_{k}=C2^{k}$, if $\epsilon <10^{-4}$ we cannot simultaneously have*

$$P_{q}\left(B_{k}\right)<\epsilon \;\mathrm{and}\;P_{q}\left(B_{k}^{\prime }\right)<\epsilon .$$

**Proof.**  Please note that this choice of ϵ means that ϵ<η/2, where η is as in Lemma 2. Recall the pivotal event $A_{k}$, and assume, for the sake of contradiction, that both probability bounds $P(B_{k})<\epsilon$ and $P(B_{k}^{\prime })<\epsilon$ hold. Please note that if $B_{k}^{c}$ holds, it means that

(7) $${\hat{M}}_{n_{k}}/M_{n_{k}}\in \left(1-\epsilon ,1+\epsilon \right),$$

and similarly if $B_{k}^{\prime c}$ holds, it means that

(8) $${\hat{M}}_{n_{k}}^{\prime }/M_{n_{k}}^{\prime }\in \left(1-\epsilon ,1+\epsilon \right).$$

By making our hypothesis, we are asserting that these events have high probabilities, 1−ϵ, under both $p_{\theta }$ and $p_{\theta^{\prime }}$ distributions, and that thus the estimator is effectively (1±ϵ)-close to the true value of the missing mass. Yet, we know that this would be violated under the pivotal event, which occurs with positive probability. We now formalize this contradiction.

By Lemma 2, we have that:

(9) $$\left.\begin{array}{cc} P(B_{k}|A_{k}) & =\frac{P(A_{k}\cap B_{k})}{P(A_{k})}\leq \frac{P(B_{k})}{P(A_{k})}\leq \frac{\epsilon }{\eta }\\ P(B_{k}^{\prime }|A_{k}) & =\frac{P(A_{k}\cap B_{k}^{\prime })}{P(A_{k})}\leq \frac{P(B_{k}^{\prime })}{P(A_{k})}\leq \frac{\epsilon }{\eta } \end{array}\;\;\right\}\;\Rightarrow \;\;P\left(B_{k}^{c}\cap B_{k}^{\prime c}|A_{k}\right)\geq 1-2\frac{\epsilon }{\eta }>0,$$

where the last inequality is strict, by the choice of ϵ<η/2.

On the other hand, recall that by Lemma 1 under $A_{k}$ we have:

$${\hat{M}}_{n_{k}}={\hat{M}}_{n_{k}}^{\prime }\;\mathrm{and}\;\frac{M_{n_{k}}}{M_{n_{k}}^{\prime }}=\frac{2-\beta }{1+\beta }=\frac{7}{5}.$$

By combining this with Equations (7) and (8), we can now see that if $\frac{1+\epsilon }{1-\epsilon }<\frac{7}{5}$, which is satisfied by any choice of ϵ<1/6, in particular ours, then if $B_{k}^{c}$ occurs, then $B_{k}^{\prime }$ occurs, and conversely if $B_{k}^{\prime c}$ occurs then $B_{k}$ occurs. For example, say $B_{k}^{c}$ occurs, then ${\hat{M}}_{n_{k}}/M_{n_{k}}<\left(1+\epsilon \right)$:

$$\frac{{\hat{M}}_{n_{k}}^{\prime }}{M_{n_{k}}^{\prime }}=\frac{{\hat{M}}_{n_{k}}}{\frac{7}{5}M_{n_{k}}}<\frac{5}{7}\left(1+\epsilon \right)<1-\epsilon ,$$

implying that Equation (8) is not satisfied, thus $B_{k}^{\prime }$ occurs. The end result is that under event $A_{k}$, $B_{k}^{c}$ and $B_{k}^{\prime c}$ cannot occur at the same time, and thus:

$$P(B_{k}^{c}\cap B_{k}^{\prime c}|A_{k})=0.$$

This contradicts the bound in (9), and establishes the lemma. □

## 3. Discussions

### 3.1. Weak Versus Strong Distribution-Free Learning

Arguably, a more common notion of learning is a strong version of Definition 1, where the sample complexity is a function of the distribution class rather than the instance. Formally:

**Definition** **4.** *We say that an estimator* learns *the missing mass in relative error* strongly *with respect to a family P of distributions, if for every ϵ>0,δ∈(0,1) there exists $n_{0}\left(P,\epsilon ,\delta \right)$ such that for all p∈P and all $n>n_{0}\left(P,\epsilon ,\delta \right)$:*

$$P_{p}\left\{\left|\frac{{\hat{M}}_{n}\left(X_{1},\cdots ,X_{n}\right)}{M_{n}\left(X_{1},\cdots ,X_{n}\right)}-1\right|<\epsilon \right\}>1-\delta .$$

*The learning is said to be* strongly distribution-free, *if P consists of* all *distributions on $N_{+}$.*

The distinction here is similar to that of uniform versus pointwise convergence. Clearly, the existence of a strong learner implies the existence of a weak learner. Conversely, as we have shown that there is no weakly distribution-free learner, there is also no strongly distribution-free learner. However, the ability to choose a different distribution at every sample size *n* makes it very easy to show this corollary directly. For example, we can consider two distributions *p* and *q* with $p\left(1\right)=1-\frac{1}{n^{2}}$ and $q\left(1\right)=1-\frac{1}{100n^{2}}$, both of which would result with overwhelming probability in a length-*n* sequence consisting entirely of this first symbol. Thus any estimator would need to predict the same missing mass with high probability. However, the rest of the symbols would have probabilities differing by a factor of 100 between the two models, and thus any estimator would be misguided for at least one of the two cases.

The relevance of the current contribution is rooted in the plausible yet misguided optimism that although we may not do well in such a worst-case paradigm, there is more hope if we first fix the instance and then study asymptotics. Our “no free lunch” theorem indeed shows the more subtle fact that there are always bad instances for every estimator, and thus even such weak learning is fundamentally impossible.

Such a contrast between weak/strong learning has also been appreciated in the classical learning literature, notably in the work of Antos and Lugosi [8]. The notions there are framed in the negative, which is why the weak/strong terminology is reversed. A traditional minimax lower bound in that context states that for any sequence of concept learners ${\hat{g}}_{n}$ at each *n* we can find a distribution for which the expected cumulative classification error is lower bounded by the complexity of the concept class. Analogously, *not* being able to strong learn as in Definition 4 means that for any estimator ${\hat{M}}_{n}$ at each *n* we can find a distribution for which the relative error stays away from zero. By demanding a *strong* performance from a learner/estimator, we are able to give only a *weak* guarantee. In particular, it could be too loose for a fixed distribution that doesn’t vary with *n*. [8] contributes by giving lower bounds that hold infinitely often for any sequence of concept learners ${\hat{g}}_{n}$ but for a distribution choice that is adversarial in advance, fixed for all *n*. Analogously, *not* being able to (weak) learn as in Definition 1 means that for any estimator ${\hat{M}}_{n}$ there exists a distribution, fixed for all *n*, for which the relative error stays away from zero for infinitely many *n*. The lower bounds of Antos and Lugosi [8] can now be tighter, which is why they call their results *strong* minimax lower bounds. In the context of the present paper, of course, the lower bounds correspond to the impossibility result, which is thus stronger since it doesn’t even hold for a fixed distribution.

### 3.2. Generalization to Continuous Tails

A closely related problem to learning the missing mass is that of estimating the tail of a probability distribution. In the simplest setting, the data consists of $Y_{1},\cdots ,Y_{n}$ that are i.i.d. samples from a continuous distribution on R. Let *F* be the cumulative distribution function. The task in question is that of estimating the tail probability

$$W_{n}=1-F\left(\max_{i=1}^{n}\;Y_{i}\right),$$

that is the probability that a new sample exceeds the maximum of all samples seen in the data.

One can immediately see the similarity with the missing mass problem, as both problems concern estimating probabilities of underrepresented events. We can use essentially the same learning framework given by Definition 1, and prove a completely parallel impossibility result.

**Theorem** **2.** *There exists ϵ>0 and a subsequence ${\left(n_{k}\right)}_{k=1,2,\cdots }$, such that for every estimator ${\hat{W}}_{n}$ of $W_{n}$ there exists a continuous distribution $F^{⋆}$, such that for all k:*

$$P_{F^{⋆}}\left\{\left|\frac{{\hat{W}}_{n_{k}}}{W_{n_{k}}}-1\right|>\epsilon \right\}>\epsilon .$$

*In particular, it follows that it is impossible to perform distribution-free learning of the tail probability in relative error.*

**Proof.**  (Sketch) The discrete version of this theorem is a trivial extension of Theorem 1, since in the proof of the latter the pivotal event forced the missing mass to be a tail probability. The potential strengthening of Theorem 2 comes from insisting on a continuous $F^{⋆}$. The same techniques may be adapted in this case, such as by dithering an exponential distribution, where a base exponential density is divided into intervals, and mass is moved between pairs of adjacent intervals by scaling the density the same way as β dithers the geometric. The adversarial distribution for a given estimator can then be chosen from this family. In order not to repeat the same aguments, however, we instead prove this result via a reduction. The details can be found in the Appendix A. Namely, we show that discrete tail estimation can be reduced to continuous tail estimation. Since the former is impossible, so is the latter. □

Theorem 2 gives a concrete justification of why it is important to make regularity assumptions when extrapolating distribution tails. This is of course the common practice of extreme value theory, see, for example [9]. Some impossibility results concerning the even more challenging problem of estimating the density of the maximum were already known [10], but to the best of our knowledge this is the first result asserting it for tail probability estimation as well.

### 3.3. Learning in Various Families

Ben-Hamou et al. [6] (Corollary 5.3) gives a very clean characterization of a sufficient learnable family, which encompasses the one covered by Ohannessian and Dahleh [5].

**Theorem** **3** **([6]).** *Denote the expected number of single-occurrence and double-occurrence symbols by $\Phi_{n,1}$ and $\Phi_{n,2}$ respectively:*

$$\Phi_{n,1}:=E\left[\sum_{x\in N_{+}}1\left\{n{\hat{p}}_{n}\left(x\right)=1\right\}\right]=\sum_{x\in N_{+}}np\left(x\right){\left[1-p\left(x\right)\right]}^{n-1},\mathrm{and}$$

$$\Phi_{n,2}:=E\left[\sum_{x\in N_{+}}1\left\{n{\hat{p}}_{n}\left(x\right)=2\right\}\right]=\sum_{x\in N_{+}}\frac{n(n-1)}{2}p{\left(x\right)}^{2}{\left[1-p\left(x\right)\right]}^{n-2}.$$

*Let H be the family defined by:*

$$H:=\left\{p\;:\;\Phi_{n,1}\to \infty \;\mathrm{and}\;\frac{\Phi_{n,2}}{\Phi_{n,1}}\mathrm{remains}\;\mathrm{bounded}\;\mathrm{as}\;n\to \infty \right\}.$$

*The Good–Turing estimator learns the missing mass in relative error with respect to H.*

The proof relies on power moment concentration inequalities (such as Chebyshev’s). The $\Phi_{n,1}\to \infty$ property embodies the heavy-tailed nature, since it says that rare events occur often. The condition that $\frac{\Phi_{n,2}}{\Phi_{n,1}}$ (i.e., its lim sup) remains bounded is a smoothness condition, since $\Phi_{n,2}$ roughly captures the variance of the number of singletons (see [6], Proposition 3.3). For us, this is instructive because one could readily verify that the condition of Theorem 3 fails for geometric (and dithered geometric) distributions. We can thus see that in some sense Good–Turing captures a maximal family of learnable distributions. In particular, we now know that the complement of H is not learnable.

Considering how sparse the dithered geometric family is, the failure of any estimator to learn the missing mass with respect to it may seem discouraging. (Please note that Theorem 1 holds even if the estimator *is aware* that this is the class it is paired with.) However, if we restrict ourselves to smooth parametric families within light tails then the outlook can be brighter. We illustrate this with the case of the geometric family.

**Theorem** **4.** *Let G be the class of geometric distributions, parametrized by α∈(0,1):*

$$p_{\alpha }\left(x\right)=\left(1-\alpha \right)\alpha^{x-1},\;\mathrm{for}x\in N_{+}.$$

*Let ${\hat{\alpha }}_{n}=1-\frac{n}{\sum X_{i}}$ be the maximum likelihood estimator of the parameter, and define the plug-in estimator:*

$${\overset{ˇ}{M}}_{n}=\sum_{x\in N_{+}}\left(1-{\hat{\alpha }}_{n}\right){\hat{\alpha }}_{n}^{x}1\left\{n{\hat{p}}_{n}\left(x\right)=0\right\}$$

*Then ${\overset{ˇ}{M}}_{n}$ learns the missing mass in relative error with respect to G.*

**Proof.**  (Sketch) The proof consists of pushing forward the convergence of the parameter to that of the entire distribution using continuity arguments, and then specializing to the missing mass. The details can be found in the Appendix B. □

### 3.4. Learning the Missing Mass in Additive Error and Learning Other Related Quantities

As mentioned in the introduction, a good part of the work on learning the missing mass focused on additive error [2,3,4]. Recently, minimax lower bounds were given for the additive error in [11] and [12]. Note however that relative error bounds cannot be deduced from these (nor any other way, given the impossibility established here.) A related problem to learning the missing mass in relative error is that of learning a distribution in KL-divergence loss. This averages all log-relative errors (missing or otherwise). This averaging scales the log-relative errors by the rare probability and attenuates the kind of gaps discussed in our present context. One thus hopes to have more optimistic results. Indeed, the Good–Turing estimator was recently shown to be adaptive/competitive for distribution learning in KL-divergence [13]. A similar result in the context of distribution learning in total variation was given in [14]. In the language of Section 3.2, being competitive can be understood as an intermediate characterization between weak and strong learning. Lastly, one could be interested in learning other properties of distributions that are intimately related to the rare component. Entropy and support size are two of these. In [15] a traditional minimax bound was established for these quantities, which was then further distilled in [16]. Another related problem is predicting the growth of the support as more observations are made. This was characterized very precisely in [17], where one can find further pointers on this very old problem. Some of these results may give the impression that nothing further can be gained from structural assumptions. However, rates can generally be refined whenever such structure exists. See for example [18] for refined competitive rates in distribution estimation and [19] for similar results in the predictive/compression setting. These results use tail characterizations, similar to those in extreme value theory [10].

### 3.5. *N*-Gram Models and Bayesian Perspectives

One of the prominent applications of estimating the missing mass has been to computational linguistics. In that context, it is known as *smoothing* and is used to estimate *N*-gram transition probabilities. The importance of accurately estimating the missing mass, and in particular in a relative-error sense, comes from the fact that *N*-grams are used to score test sentences using log-likeliehoods. Test sentences often have transitions that are never seen in the training corpus, and thus in order for the inferred log-likelihoods to accurately track the true log-likelihood, these rare transitions need to be assigned meaningful values, ideally as close to the truth as possible. As such, various forms of smoothing, including Good–Turing esimation, have become an essential ingredient of many practical algorithms, such as the popular method proposed by Kneser and Ney [20].

In the context of *N*-gram learning, a separate Bayesian perspective was also proposed. One of the earliest to introduce this were [21] using a Dirichlet prior. This was shown to not be very effective, and we now understand that it is due to the fact that (1) the Dirichlet process produces light tails while language is often heavy-tailed and, even if it were; (2) rare probabilities are hard to learn for large light-tailed families. The natural progression of these Bayesian models led to the use of the two-parameter Poisson-Dirichlet prior [22], which was suggested initially by [23]. Despite employing sophisticated inference techniques, the missing mass estimator that resulted from these models closely followed the Good–Turing estimator (for a sharp analysis of this correspondence, see Falahatgar et al. [18].) In light of the present work, this is not surprising since the two-parameter Poisson-Dirichlet process almost surely produces heavy-tailed distributions, and any two algorithms that learn the missing mass are bound to have the same qualitative behavior.

## 4. Summary

In this paper, we have considered the problem of learning the missing mass, which is the probability of all unseen symbols in an i.i.d. draw from an unknown discrete distribution. We have phrased this in the probabilistic framework of learning. Our main contribution was to show that it is not possible to learn the missing mass in a completely distribution-free fashion.

In other words, no single estimator can do well for all distributions. We have given a detailed account of the proof, emphasizing the intuition of how failure can occur in large light-tailed families. We have also placed this work in a greater context, through some discussions and extensions of the impossibility result to continuous tail probability estimation, and by showing that smaller, parametric, light-tailed families may be learnable.

An initial impetus for this paper and its core message is that assuming further structure can be necessary in order to learn rare events. Further structure, of course, is nothing more than a form of regularization. This is a familiar notion to the computational learning community, but for a long time the Good–Turing estimator enjoyed favorable analysis that focused on additive error, and evaded this kind of treatment. The essential ill-posedness of the problem was uncovered by studying relative error. But lower bounds cannot be deduced from the failure of particular algorithms. Our result thus completes the story, and we can now shift our attention to studying the landscape that is revealed.

The most basic set of open problems concerns establishing families that allow learning of the missing mass. We have seen in this paper some such families, including the heavy-tailed family learnable by the Good–Turing estimator, and simple smooth parametric families, learnable using plug-in estimators. How do we characterize such families more generally? The next layer of questions concerns establishing convergence rates, i.e., strong learnability, via both lower and upper bounds. The fact that a family of distributions allows learning does not mean that such rates can be established. This is because any estimator may be faced with arbitrarily slow convergence, by varying the distribution in the family. In other words we may be faced with a lack of uniformity. How do we control the convergence rate? Lastly, when learning is not possible, we may want to establish how gracefully an estimator can be made to fail. Understanding these limitations and accounting for them can be critical to the proper handling of data-scarce learning problems.

## Appendix A. This Appendix Presents the Proof of Theorem 2

Let us first consider the tail estimation problem in the discrete case. $W_{n}$ is still well-defined here. But in order to clearly delineate the discrete case, let us define/rename the tail probability as:

(A1) $$T_{n}:=\sum_{x>\max_{i}X_{i}}p\left(x\right),$$

and let ${\hat{T}}_{n}$ denote an arbitrary estimator of $T_{n}$. It is immediate that $T_{n}$ is identical to $W_{n}$ in the discrete case. Next, note that in the proof of Theorem 1, the pivotal event $A_{k}$ given by Equation (6) forces the missing mass to be a tail probability (see the proof of Lemma 1.) That is, under $A_{k}$, $T_{n_{k}}=M_{n_{k}}$. It therefore follows that Lemma 1 holds with $M_{n}$ and ${\hat{M}}_{n}$ replaced by $T_{n}$ and ${\hat{T}}_{n}$ respectively. Lemma 2 only depends on the definition of the pivotal event, not the missing mass. And the argument of Lemma 3 follows exactly identically with again $M_{n}$ and ${\hat{M}}_{n}$ replaced by $T_{n}$ and ${\hat{T}}_{n}$ respectively everywhere in the statement and its proof. Consequently, the proof of Theorem 1 produces the following parallel result. There exist γ>1 and a sequence ${\left(n_{k}\right)}_{k=1,2,\cdots }$ (both universal) such that for every ${\hat{T}}_{n}$ we can find a distribution $p^{⋆}$ (which does depend on ${\hat{T}}_{n}$), such that for all *k* (a slight notational change):

(A2) $$P_{p^{⋆}}\left\{\frac{{\hat{T}}_{n_{k}}}{T_{n_{k}}}\notin \left[\gamma^{-1},\gamma \right]\right\}>\gamma -1.$$

So essentially, Theorem 2 is a direct corollary of our main result if we allow any distribution on the real line, including discrete ones. However, we want to show that the same result holds even over the family of properly continuous distributions, which have a density on R with respect to the Lebesgue measure (if the density is not otherwise restricted). The proof of this fact could be done by paralleling the proof thus far, but by dithering a continuous distribution instead. However, for the sake of novelty, we prove it instead via reduction from discrete to continuous.

We first make a minor generalization of the discrete framework. Observe that nowhere in the proof of Theorem 1 was ${\hat{M}}_{n}$ required to be a deterministic function of the observation. So ${\hat{M}}_{n}$ and ${\hat{T}}_{n}$ could be *randomized*, i.e., depend on an additional random element $\zeta_{n}$ that is independent of the observation and any of the parameters of the problem (see Definition 1.) More rigorously, we include this randomness in the coupling of the proof by simply letting $\zeta_{n}=\zeta_{n}^{\prime }$ always. This way, the samples being identical still implies that the values of the estimators are identical. This is the only property of the estimator we used and thus all the arguments follow.

The rest of the proof basically reduces the ability to learn the discrete tail with a randomized estimator to the ability to learn the continuous tail. The claimed impossibility follows from this reduction. Let us thus assume, for the sake of contradiction, that there exists ${\hat{W}}_{n}$ s.t. for all continuous distributions *F*, for all η>1:

(A3) $$P_{F}\left\{\frac{{\hat{W}}_{n}}{W_{n}}\in \left[\eta^{-1},\eta \right]\right\}\to 0,\mathrm{as}n\to \infty .$$

Next, recall that by construction we more precisely know that $p^{⋆}$ from earlier can be chosen from the $\left(\beta =\frac{1}{4},m=1\right)$-dithered geometric$\left(\frac{1}{2}\right)$ family. It is easy to check that for all such distributions, the tail is comparable to its preceding symbol’s probability. In particular it is not much smaller: there exists κ<∞ such that for all *p* in this family

$$\max_{x}\frac{p(x)}{\sum_{x^{\prime }>x}p\left(x^{\prime }\right)}\leq \kappa ,$$

and we can always choose $\kappa >\sqrt{\gamma }-1$ (by capping from below). For reasons that will shortly become apparent, choose any *m* such that

$${\left(1-\frac{\sqrt{\gamma }-1}{\kappa }\right)}^{m}<\frac{\gamma -1}{2}.$$

Now let *G* be any continuous distribution on [0,1]. Denote by $\bar{G}:=1-G$ the corresponding tail probability function and let ${\bar{G}}^{-1}\left(t\right):=\inf\left\{z\;:\;\bar{G}\left(z\right)\leq t\right\}$ denote its left inverse. Note two properties: $\bar{G}\left(z\right)>t$ and $z<{\bar{G}}^{-1}\left(t\right)$ are equivalent, and $\bar{G}\left({\bar{G}}^{-1}\left(t\right)\right)=t$.

Let $Z_{i,\ell }$ be i.i.d. draws from *G*, for i=1,⋯,n and ℓ=1,⋯,m. Let $\zeta_{n}={\left(Z_{i,\ell }\right)}_{i=1,\cdots ,n;\;\ell =1,\cdots ,m}$, and for a given observation sequence $x_{1},\cdots ,x_{n}$ in $N_{+}^{n}$, define the randomized estimator:

(A4) $${\hat{T}}_{n}\left(x_{1},\cdots ,x_{n},\zeta_{n}\right):=\min_{\ell =1}^{m}{\hat{W}}_{n}\left(x_{1}+Z_{1,\ell },\cdots ,x_{n}+Z_{n,\ell }\right).$$

For this specific choice of estimator, let $p^{⋆}$ be the distribution that yields the impossibility result in Equation (A2). Let $X_{i}$ be i.i.d. drawn from $p^{⋆}$, independently of the infinite array $\left(Z_{i,\ell }\right)$. Observe that then, for each *ℓ*, the sequence $Y_{i,\ell }:=X_{i}+Z_{i,\ell }$ is i.i.d. distributed according to a *continuous* distribution $F^{⋆}$, and has its own tail probability $W_{n,\ell }:=1-F^{⋆}\left(\max_{i}Y_{i,\ell }\right)$. Also, let the estimate of this tail probability for each *ℓ* be denoted by ${\hat{W}}_{n,\ell }:={\hat{W}}_{n}\left(Y_{1,\ell },\cdots ,Y_{n,\ell }\right)$. Accordingly, based on our assumption, the limiting property in Equation (A3) holds and for any η>1:

$$\forall \ell \;P\left\{\frac{{\hat{W}}_{n,\ell }}{W_{n,\ell }}\notin \left[\eta^{-1},\eta \right]\right\}\to 0,\mathrm{as}n\to \infty .$$

Please note that if for all *ℓ* we have $\eta^{-1}<\frac{a_{\ell }}{b_{\ell }}<\eta$ then also $\eta^{-1}<\frac{\min_{\ell }a_{\ell }}{\min_{\ell }b_{\ell }}<\eta$. Thus:

(A5) $$\begin{array}{ccc} P\left\{\frac{\min_{\ell }{\hat{W}}_{n,\ell }}{\min_{\ell }W_{n,\ell }}\notin \left[\eta^{-1},\eta \right]\right\} & \leq & P\overset{m}{\underset{\ell =1}{⋃}}\left\{\frac{{\hat{W}}_{n,\ell }}{W_{n,\ell }}\notin \left[\eta^{-1},\eta \right]\right\}\\ & \leq & m\;P\left\{\frac{{\hat{W}}_{n,1}}{W_{n,1}}\notin \left[\eta^{-1},\eta \right]\right\}\to 0,\mathrm{as}n\to \infty . \end{array}$$

This means that $\min_{\ell }{\hat{W}}_{n,\ell }\equiv {\hat{T}}_{n}$ (recall the definition of Equation (A4)) is a good estimator of $\min_{\ell }W_{n,\ell }$. The contradiction we’re after is to show that ${\hat{T}}_{n}$ thus defined is also a good estimator of $T_{n}$ (given by Equation (A1) with $p=p^{⋆}$), so we need to compare $\min_{\ell }W_{n,\ell }$ and $T_{n}$ and show that they are close.

Let us first fix one instance of *ℓ*. For clarity, let us momentarily drop the *ℓ*-notation from subscripts. For one fixed such instance, note that $W_{n}$ is a continuous tail that is coupled with $T_{n}$: they are related through the common values of *X*. For notational convenience, let $X_{\max}:=\max_{i}X_{i}$ and let $Y_{\max}=\max_{i}Y_{i}$. Now observe that $T_{n}$ is equal to $\sum_{x>X_{\max}}p\left(x\right)$ and it is exactly the $F^{⋆}$-measure of the interval $\left[X_{\max}+1,\infty \right)$, by construction. On the other hand $W_{n}$ is the $F^{⋆}$-measure of the interval $\left[Y_{\max},\infty \right)$. Since $Y_{\max}\in \left[X_{\max},X_{\max}+1\right]$, it follows that $W_{n}\geq T_{n}$. How much larger can it get? By at most $p(X_{\max})$. More formally, we can write $F^{⋆}\left(x\right)=\sum_{x^{\prime }}p\left(x^{\prime }\right)G\left(x-x^{\prime }\right)$. Thus $W_{n}-T_{n}=\int_{Y_{\max}}^{X_{\max}+1}F^{⋆}\left(dx\right)=\sum_{x^{\prime }}p\left(x^{\prime }\right)\int_{Y_{\max}-x^{\prime }}^{X_{\max}+1-x^{\prime }}G\left(dx\right)=p\left(X_{\max}\right)\bar{G}\left(Y_{\max}-X_{\max}\right)$, since the inner integral is non-zero only for $x^{\prime }=X_{\max}$. Define the (random) set of maximizing observations as $I_{\max}=\left\{i:X_{i}=X_{\max}\right\}$. Now, let us bound the probability of any particular excess beyond $p(X_{\max})$ times a factor t∈(0,1):

(A6) $$\begin{array}{ccc} P\{W_{n}>T_{n}+p\left(X_{\max}\right)\cdot t\} & = & P\left\{\bar{G}\left(Y_{\max}-X_{\max}\right)>t\right\}=P\left\{Y_{\max}-X_{\max}<{\bar{G}}^{-1}\left(t\right)\right\}\\ & = & P\left\{\forall i\in I_{\max},\;Z_{i}<{\bar{G}}^{-1}\left(t\right)\right\}\\ & = & E\left[P\left(\left.{⋂}_{i\in I_{\max}}\left\{Z_{i}<{\bar{G}}^{-1}\left(t\right)\right\}\;\right|X_{1},\cdots ,X_{n})\right)\right]\\ & = & E\left[P^{|I_{\max}|}\left\{Z<{\bar{G}}^{-1}\left(t\right)\right\}\right]\\ & \leq & P\left\{Z\leq {\bar{G}}^{-1}\left(t\right)\right\}=G\left({\bar{G}}^{-1}\left(t\right)\right)=1-t, \end{array}$$

where we conditioned over all $X_{i}$, used the independence of the $Z_{i}$’s from the $X_{i}$’s to write the probability of the intersection as a product, used the fact that the $Z_{i}$ have identical distribution to a generic *Z*, and finally bounded $|I_{\max}|$ (the only term still depending on the $X_{i}$’s and thus influence by the outer expectation) by 1. The only loss here is from this replacement. This, however, can be shown to be rather tight. For intuition, note that a geometric-$\frac{1}{2}$ has only a single maximizing observation in expectation, i.e., $E[|I_{\max}|]=1$. This is not good news, since $p\left(X_{\max}\right)/T_{n}=\frac{p(X_{\max})}{\sum_{x>X_{\max}}p\left(x\right)}$ is lower bounded away from zero in the dithered geometric family, and thus this shows that we cannot expect $T_{n}$ to be arbitrarily close to a single $W_{n}$ with probability that is arbitrarily large. This is true *regardless* of the choice of *G*. This is the motivation behind choosing the smallest of *m* continuous versions for the reduction, which restores the needed maneuverability for the approximation. Indeed, now restoring the *ℓ*-notation:

(A7) $$\begin{array}{ccc} P\{\min_{\ell =1}^{m}W_{n,\ell }>T_{n}+p\left(X_{\max}\right)\cdot t\} & = & P{⋂}_{\ell =1}^{m}\left\{W_{n,\ell }>T_{n}+p\left(x\right)\cdot t\right\}\\ & = & P{⋂}_{\ell =1}^{m}\left\{\forall i\in I_{\max},\;Z_{i,\ell }<{\bar{G}}^{-1}\left(t\right)\right\}\\ & = & E\left[P\left(\left.{⋂}_{\ell =1}^{m}{⋂}_{i\in I_{\max}}\left\{Z_{i,\ell }<{\bar{G}}^{-1}\left(t\right)\right\}\;\right|X_{1},\cdots ,X_{n}\right)\right]\\ & = & E\left[P^{m|I_{\max}|}\left\{Z<{\bar{G}}^{-1}\left(t\right)\right\}\right]\\ & \leq & P^{m}\left\{Z\leq {\bar{G}}^{-1}\left(t\right)\right\}=G{\left({\bar{G}}^{-1}\left(t\right)\right)}^{m}={\left(1-t\right)}^{m}, \end{array}$$

where the only notable observation is that the *m* replicated versions compound the number of *Z*s that deviate. The rest is derived in the same way as in Equation (A6). Finally, using the fact that $\frac{p(X_{\max})}{T_{n}}\leq \max_{x}\frac{p(x)}{\sum_{x^{\prime }>x}p\left(x^{\prime }\right)}\leq \kappa$, we have:

$$P\left\{\frac{\min_{\ell =1}^{m}W_{n,\ell }}{T_{n}}>1+\kappa t\right\}\leq {\left(1-t\right)}^{m}.$$

Specializing to $1+\kappa t=\sqrt{\gamma }$, using the fact that we chose *m* such that ${\left(1-\frac{\sqrt{\gamma }-1}{\kappa }\right)}^{m}<\frac{\gamma -1}{2}$, and recalling that the ratio cannot be less than 1, we have for all *n* that:

(A8) $$P\left\{\frac{\min_{\ell =1}^{m}W_{n,\ell }}{T_{n}}\notin \left[{\sqrt{\gamma }}^{-1},\sqrt{\gamma }\right]\right\}<\frac{\gamma -1}{2}.$$

Also specializing Equation (A5) to $\eta =\sqrt{\gamma }$, we have that for *n* large enough:

(A9) $$P\left\{\frac{\min_{\ell =1}^{m}W_{n,\ell }}{{\hat{T}}_{n}}\notin \left[{\sqrt{\gamma }}^{-1},\sqrt{\gamma }\right]\right\}\leq \frac{\gamma -1}{2}.$$

By combining these two approximations, our reduction is complete. Namely, given our assumption (A3) that we can estimate the continuous tail, we have that for *n* large enough we can estimate the discrete tail to our desired accuracy:

$$P\left\{\frac{{\hat{T}}_{n}}{T_{n}}\notin \left[\gamma^{-1},\gamma \right]\right\}<\gamma -1,$$

which clearly contradicts the impossibility (A2) of estimating the discrete tail. More precisely, since the continuous vs. discrete tail approximation in Equation (A8) does *not* depend on the assumption, it must be that it’s Equation (A9) that fails for each $\left(n_{k}\right)$. Recalling the bound of Equation (A5), we must have:

$$mP\left\{\frac{{\hat{W}}_{n_{k}}}{W_{n_{k}}}\notin \left[{\sqrt{\gamma }}^{-1},\sqrt{\gamma }\right]\right\}\geq P\left\{\frac{\min_{\ell }W_{n_{k},\ell }}{{\hat{T}}_{n_{k}}}\notin \left[{\sqrt{\gamma }}^{-1},\sqrt{\gamma }\right]\right\}>\frac{\gamma -1}{2}.$$

Finally, set $\epsilon =\min\left\{\sqrt{\gamma }-1,1-{\sqrt{\gamma }}^{-1},\frac{\gamma -1}{2m}\right\}$ with the above, to obtain the exact claim of the theorem. Namely, for this absolute constant ϵ and subsequence $\left(n_{k}\right)$, given any ${\hat{W}}_{n}$ we can construct $F^{⋆}$ as we did (with an arbitrary *G*), such that $P_{F^{⋆}}\left\{\frac{{\hat{W}}_{n_{k}}}{W_{n_{k}}}\notin \left[1-\epsilon ,1+\epsilon \right]\right\}>\epsilon$ for all *k*.

## Appendix B. This Appendix Presents the Proof of Theorem 4

### Appendix B.1. Notation and Outline

Let us first set some notation. Recall that the mean of the geometric distribution $p_{\alpha }\left(x\right)=\left(1-\alpha \right)\alpha^{x-1}$ is $\mu =\frac{1}{1-\alpha }$ and its variance is $\sigma^{2}=\frac{\alpha }{{\left(1-\alpha \right)}^{2}}$. Let us write the empirical mean and our parameter estimate respectively as follows:

$${\hat{\mu }}_{n}=\frac{1}{n}\sum_{i=1}^{n}X_{i},\;{\hat{\alpha }}_{n}=1-\frac{1}{{\hat{\mu }}_{n}}.$$

The plug-in probability estimate can be expressed as:

$${\overset{ˇ}{p}}_{n}\left(x\right):=\left(1-{\hat{\alpha }}_{n}\right){\hat{\alpha }}_{n}^{x-1}.$$

Using our notation for the missing symbols, $E_{n}:=\left\{x\in N_{+}:\hat{p}\left(x\right)=0\right\},$ the missing mass is

$$M_{n}=p_{\alpha }\left(E_{n}\right)=\sum_{x\in E_{n}}\left(1-\alpha \right)\alpha^{x-1}$$

and the suggested plug-in estimator can be written as

$${\overset{ˇ}{M}}_{n}:={\overset{ˇ}{p}}_{n}\left(E_{n}\right)=\sum_{x\in E_{n}}\left(1-{\hat{\alpha }}_{n}\right){\hat{\alpha }}_{n}^{x-1}.$$

The following proof first establishes the convergence of the parameter estimate and then pushes it forward to the entire distribution, specializing in particular to the missing mass. For the latter, we establish some basic localization properties of the punctured segment of a geometric sample coverage. This is related to the general study of gaps, see, for example, [24].

We have the following elementary convergence property for the parameter.

**Lemma** **A1** (Parameter Convergence)**.** *Let δ>0, and define:*

$$\epsilon_{n}:=\sqrt{\frac{\alpha }{\delta n}}\cdot \left(\frac{\max\{1,\frac{1-\alpha }{\alpha }\}}{1-\sqrt{\frac{\alpha }{\delta n}}}\right).$$

*Then, at every $n>\frac{\alpha }{\delta }$, we have that with probability greater than 1−δ:*

$$\left|\frac{{\hat{\alpha }}_{n}}{\alpha }-1\right|\leq \epsilon_{n}\;\mathrm{and}\;\left|\frac{1-{\hat{\alpha }}_{n}}{1-\alpha }-1\right|\leq \epsilon_{n}.$$

*If we let $\eta_{n}=\epsilon_{n}/\left(1-\epsilon_{n}\right)$, we can also write this as*

$$\frac{1}{1+\eta_{n}}\leq \frac{{\hat{\alpha }}_{n}}{\alpha }\leq 1+\eta_{n}\;\mathrm{and}\;\frac{1}{1+\eta_{n}}\leq \frac{1-{\hat{\alpha }}_{n}}{1-\alpha }\leq 1+\eta_{n}.$$

**Proof.** From Chebyshev’s inequality, we know that for all δ>0:

$$P\left\{|{\hat{\mu }}_{n}-\mu |\leq \frac{\sigma }{\sqrt{\delta n}}\right\}\geq 1-\delta .$$

We now simply have to verify that $|{\hat{\mu }}_{n}-\mu |\leq \frac{\sigma }{\sqrt{\delta n}}$ implies that both $\left|\frac{{\hat{\alpha }}_{n}}{\alpha }-1\right|$ and $\left|\frac{1-{\hat{\alpha }}_{n}}{1-\alpha }-1\right|$ are smaller than $\epsilon_{n}$. Indeed, using ${\hat{\mu }}_{n}\geq \mu -\frac{\sigma }{\sqrt{\delta n}}$:

$$\left|\frac{{\hat{\alpha }}_{n}}{\alpha }-1\right|=\left|\frac{({\hat{\mu }}_{n}-1)\mu }{{\hat{\mu }}_{n}\left(\mu -1\right)}-1\right|=\left|\left({\hat{\mu }}_{n}-\mu \right)\frac{1}{{\hat{\mu }}_{n}\left(\mu -1\right)}\right|\leq \left|{\hat{\mu }}_{n}-\mu \right|\frac{1}{\left(\mu -\frac{\sigma }{\sqrt{\delta n}}\right)\left(\mu -1\right)}$$

and

$$\left|\frac{1-{\hat{\alpha }}_{n}}{1-\alpha }-1\right|=\left|\frac{\mu }{{\hat{\mu }}_{n}}-1\right|=\left|\left(\mu -{\hat{\mu }}_{n}\right)\frac{1}{{\hat{\mu }}_{n}}\right|\leq \left|{\hat{\mu }}_{n}-\mu \right|\frac{1}{\left(\mu -\frac{\sigma }{\sqrt{\delta n}}\right)}.$$

Finally, since $\left|{\hat{\mu }}_{n}-\mu \right|\leq \frac{\sigma }{\sqrt{\delta n}}$, both of these bounds are smaller than:

$$\frac{\sigma }{\sqrt{\delta n}}\frac{1}{\left(\mu -\frac{\sigma }{\sqrt{\delta n}}\right)\min\left\{1,\mu -1\right\}}=\frac{\frac{\sqrt{\alpha }}{1-\alpha }}{\sqrt{\delta n}}\frac{1}{\left(\frac{1}{1-\alpha }-\frac{\sqrt{\alpha }}{1-\alpha }\frac{1}{\sqrt{\delta n}}\right)\min\left\{1,\frac{\alpha }{1-\alpha }\right\}},$$

which is equal to $\epsilon_{n}$. The expression with $\eta_{n}$ follows from $1-\epsilon_{n}=\frac{1}{1+\eta_{n}}$ and $1+\eta_{n}>1+\epsilon_{n}$. □

It follows from Lemma A1 that with probability greater than 1−δ, we have the following pointwise convergence of the distribution.

$${\left(1+\eta_{n}\right)}^{-x}\left(1-\alpha \right)\alpha^{x-1}\leq {\overset{ˇ}{p}}_{n}\left(x\right)\leq {\left(1+\eta_{n}\right)}^{x}\left(1-\alpha \right)\alpha^{x-1}.$$

Since the rate of this convergence is not uniform, we need to exercise care when specializing to particular events. We focus on the missing symbols’ event. We have:

(A10) $$\frac{\sum_{x\in E_{n}}{\left(1+\eta_{n}\right)}^{-x}\left(1-\alpha \right)\alpha^{x-1}}{\sum_{x\in E_{n}}\left(1-\alpha \right)\alpha^{x-1}}\leq \frac{{\overset{ˇ}{M}}_{n}}{M_{n}}=\frac{{\overset{ˇ}{p}}_{n}\left(E_{n}\right)}{p_{\alpha }\left(E_{n}\right)}\leq \frac{\sum_{x\in E_{n}}{\left(1+\eta_{n}\right)}^{x}\left(1-\alpha \right)\alpha^{x-1}}{\sum_{x\in E_{n}}\left(1-\alpha \right)\alpha^{x-1}}.$$

The event $E_{n}$ is inconvenient to sum over, because it has points spread out randomly. This is particularly true for its initial portion, where the samples “puncture” it. It is more convenient to approximate this segment in order to bound Equation (A10). We now formalize this notion, via the following definition.

**Definition** **A1** (Punctured Segment)**.** *The punctured segment of a sample is the part between the end of the first contiguous coverage and the end of the total coverage. Its extremities are:*

$$V_{n}^{-}:=\min E_{n}\;\mathrm{and}\;V_{n}^{+}:=\max E_{n}^{c}.$$

We have the following localization property for the punctured segment of samples from a geometric distribution.

**Lemma** **A2** (Localization of Punctured Segment)**.** *Let $X_{1},\cdots ,X_{n}$ be samples from a geometric distribution $p_{\alpha }\left(x\right)=\left(1-\alpha \right)\alpha^{x-1}$ on $N_{+}$. Let $V_{n}^{-}$ and $V_{n}^{+}$ be the extremities of the punctured segment as defined in Definition A1. Then, for all $u>{\left(\frac{\alpha }{1-\alpha }\right)}^{2}$, we have:*

$$\begin{array}{cc} P\{V_{n}^{-}<\log_{1/\alpha }\left(n\right)-\log_{1/\alpha }\left(u\right)\} & <2e^{-\frac{1-\alpha }{\alpha }u}<\frac{\alpha }{(1-\alpha )u},\\ P\{V_{n}^{+}>\log_{1/\alpha }\left(n\right)+1+\log_{1/\alpha }\left(u\right)\} & <\frac{1}{u}. \end{array}$$

*In particular, for $\delta <\left(1-\alpha \right)/\alpha^{2}$, we have that with probability greater than 1−δ:*

$$\log_{1/\alpha }\left(n\right)-\log_{1/\alpha }\left[\frac{1}{(1-\alpha )\delta }\right]\leq V_{n}^{-}<V_{n}^{+}\leq \log_{1/\alpha }\left(n\right)+1+\log_{1/\alpha }\left[\frac{1}{(1-\alpha )\delta }\right].$$

**Proof.** Given an integer a≥2, the event that $V_{n}^{-}<a$ implies that at least one of the symbols below *a* did not appear in the sample. By using the union bound, we thus have that:

$$\begin{array}{ccc} P\{V_{n}^{-}<a\} & \leq & \sum_{x=1}^{a-1}{\left[1-\left(1-\alpha \right)\alpha^{x-1}\right]}^{n}\\ & = & \sum_{\ell =1}^{a-1}{\left[1-\frac{\left(1-\alpha \right)n\alpha^{a-1-\ell }}{n}\right]}^{n}\\ & \leq & \sum_{\ell =1}^{a-1}\exp\left[-\left(1-\alpha \right)n\alpha^{a-1-\ell }\right]\;\leq \;\sum_{\ell =1}^{\infty }\exp\left[-\left(1-\alpha \right)n\alpha^{a-1-\ell }\right] \end{array}$$

By specializing to $a\left(u,n\right)=\left\lfloor \log_{1/\alpha }\left(n\right)+1-\log_{1/\alpha }\left(u\right)\right\rfloor$:

$$\begin{array}{ccc} P\{V_{n}^{-}<\log_{1/\alpha }\left(n\right)-\log_{1/\alpha }\left(u\right)\} & \leq & P\{V_{n}^{-}<a\left(u,n\right)\}\\ & \leq & \sum_{\ell =1}^{\infty }\exp\left[-\left(1-\alpha \right)n\alpha^{\log_{1/\alpha }\left(n\right)-\log_{1/\alpha }\left(u\right)-\ell }\right]\\ & = & \sum_{\ell =1}^{\infty }\exp\left[-\left(1-\alpha \right)\alpha^{-\ell }u\right]. \end{array}$$

Lastly, if $u>{\left(\frac{\alpha }{1-\alpha }\right)}^{2}$, one can show by induction that $\left(1-\alpha \right)\alpha^{-\ell }u\geq \frac{1-\alpha }{\alpha }u+\ell -1$. This turns the sum into a geometric series, giving:

$$P\left\{V_{n}^{-}<\log_{1/\alpha }\left(n\right)-\log_{1/\alpha }\left(u\right)\right\}\leq e^{-\frac{1-\alpha }{\alpha }u}\sum_{\ell =1}^{\infty }e^{-\ell +1}<2e^{-\frac{1-\alpha }{\alpha }u}<\frac{\alpha }{(1-\alpha )u}.$$

Next, note that $V_{n}^{+}$ is nothing but the maximum of the samples. Thus, given an integer $b\in N_{+}$, the event $V_{n}^{+}>b$ is the complement of the event that all the samples are at *b* or below. Since the total probability of the range 1,⋯,b is $1-\alpha^{b}$, we thus have:

$$P\left\{V_{n}^{+}>b\right\}=1-{\left(1-\alpha^{b}\right)}^{n}.$$

If we now specialize to $b\left(u,n\right)=\left\lceil \log_{1/\alpha }\left(n\right)+\log_{1/\alpha }\left(u\right)\right\rceil$, we have that:

$$\begin{array}{ccc} P\{V_{n}^{+}>\log_{1/\alpha }\left(n\right)+1+\log_{1/\alpha }\left(u\right)\} & \leq & P\{V_{n}^{+}>b\left(u,n\right)\}\\ & \leq & 1-{\left(1-\alpha^{\log_{1/\alpha }\left(n\right)+\log_{1/\alpha }\left(u\right)}\right)}^{n}\\ & = & 1-{\left(1-\frac{1}{u\cdot n}\right)}^{n}<\frac{1}{u}. \end{array}$$

For the last part of the claim, we let $u=\frac{1}{(1-\alpha )\delta }$, followed by a union bound on the analyzed events. This gives us that at least one of the two events holds with probability at most $\frac{1}{u}+\frac{\alpha }{(1-\alpha )u}=\delta$, and therefore neither holds with probability at least 1−δ, as desired. □

### Appendix B.2. Completing the Proof

We now put together the pieces of the proof of Theorem 4. To show that our estimator learns the missing mass in relative error with respect to G, we obtain the following equivalent statement. Fix δ>0 and η>0. We prove that for *n* large enough with probability greater than 1−2δ we have:

$$\frac{1}{1+\eta }<\frac{{\overset{ˇ}{M}}_{n}}{M_{n}}<1+\eta .$$

Without loss of generality, to satisfy the conditions of Lemmas A1 and A2, we restrict ourselves to $\delta <\left(1-\alpha \right)/\alpha^{2}$ (we can always choose a smaller δ than specified) and $n>\frac{\alpha }{\delta }$ (we can always ask for *n* to be larger). As such, we have that with probability at least 1−2δ, both events of Lemmas A1 and A2 occur. We work under the intersection of these events.

We give the details of only the right tail of the convergence; all the steps can be directly paralleled for the left tail. To see why the punctured set is a useful notion, we claim that the following quantity upper bounds the right tail of Equation (A10):

(A11) $$\begin{array}{ccc} \frac{\sum_{x>V_{n}^{+}}{\left(1+\eta_{n}\right)}^{x}\left(1-\alpha \right)\alpha^{x-1}}{\sum_{x>V_{n}^{+}}\left(1-\alpha \right)\alpha^{x-1}} & = & {\left(1+\eta_{n}\right)}^{V_{n}^{+}}\frac{\sum_{y\in N_{+}}{\left(1+\eta_{n}\right)}^{y}\left(1-\alpha \right)\alpha^{y-1}}{\sum_{y\in N_{+}}\left(1-\alpha \right)\alpha^{y-1}=1}\\ & = & {\left(1+\eta_{n}\right)}^{V_{n}^{+}}\frac{\left(1-\alpha \right)\left(1+\eta_{n}\right)}{1-\alpha (1+\eta_{n})}. \end{array}$$

where for the first equality we have used the change of variable $y=x-V_{n}^{+}$ and simplified the common α factors in the numerator and denominator, and for the second equality we have used the moment generating function of the geometric distribution: $E\left[e^{sX}\right]=\left(1-\alpha \right)e^{s}/\left(1-\alpha e^{s}\right)$. To prove this claim, we proceed by induction, starting at step t=1 with the set $G^{\left(1\right)}:=\left\{V_{n}^{+}+1,V_{n}^{+}+2,\cdots \right\}\subset E_{n}$, adding at every step *t* the largest element $z^{\left(t\right)}$ of $E_{n}$ not yet in $G^{\left(t-1\right)}$ to obtain $G^{\left(t\right)}$, and proving that:

$$\frac{\sum_{x\in G^{\left(t\right)}}{\left(1+\eta_{n}\right)}^{x}\left(1-\alpha \right)\alpha^{x-1}}{\sum_{x\in G^{\left(t\right)}}\left(1-\alpha \right)\alpha^{x-1}}\leq \frac{\sum_{x\in G^{\left(t-1\right)}}{\left(1+\eta_{n}\right)}^{x}\left(1-\alpha \right)\alpha^{x-1}}{\sum_{x\in G^{\left(t-1\right)}}\left(1-\alpha \right)\alpha^{x-1}}.$$

We use the following basic property that for positive real numbers $a_{1},b_{1},a_{2},b_{2}$, the following three equalities are equivalent (these are *mediant inequalities*):

$$\begin{array}{cccc} \left(i\right) & \;a_{1}/b_{1} & \leq & a_{2}/b_{2},\\ \left(ii\right) & \;a_{1}/b_{1} & \leq & \left(a_{1}+a_{2}\right)/\left(b_{1}+b_{2}\right),\\ \left(iii\right) & \;\left(a_{1}+a_{2}\right)/\left(b_{1}+b_{2}\right) & \leq & a_{2}/b_{2}. \end{array}$$

For the base case, let $a_{2}=\sum_{x\in G^{\left(1\right)}}{\left(1+\eta_{n}\right)}^{x}\left(1-\alpha \right)\alpha^{x-1}$ and $b_{2}=\sum_{x\in G^{\left(1\right)}}\left(1-\alpha \right)\alpha^{x-1}$. We then choose the largest $z^{\left(1\right)}\in E_{n}\setminus G^{\left(1\right)}$ and we let $a_{1}={\left(1+\eta_{n}\right)}^{z^{\left(1\right)}}\left(1-\alpha \right)\alpha^{z^{\left(1\right)}-1}$ and $b_{1}=\left(1-\alpha \right)\alpha^{z^{\left(1\right)}-1}$. From (A11), noting that the fraction is always greater than 1, it follows that $a_{2}/b_{2}>{\left(1+\eta_{n}\right)}^{V_{n}^{+}}>{\left(1+\eta_{n}\right)}^{z^{\left(1\right)}}=a_{1}/b_{1}$. We can thus add $z^{\left(1\right)}$ to the sum, and obtain $\left(a_{1}+a_{2}\right)/\left(b_{1}+b_{2}\right)\leq a_{2}/b_{2}$, establishing the base case. Please note that this also shows that $\left(a_{1}+a_{2}\right)/\left(b_{1}+b_{2}\right)\geq a_{1}/b_{1}={\left(1+\eta_{n}\right)}^{z^{\left(1\right)}}$. We pass this property down by induction, and we can assume this holds true at every step.

To continue the induction at step *t*, let $a_{2}=\sum_{x\in G^{\left(t-1\right)}}{\left(1+\eta_{n}\right)}^{x}\left(1-\alpha \right)\alpha^{x-1}$ and $b_{2}=\sum_{x\in G^{\left(t-1\right)}}\left(1-\alpha \right)\alpha^{x-1}$. As noted, we assume that $a_{2}/b_{2}\geq {\left(1+\eta_{n}\right)}^{z^{\left(t-1\right)}}$ from the previous induction step. We then choose the largest $z^{\left(t\right)}\in E_{n}\setminus G^{\left(t-1\right)}$ and we let $a_{1}={\left(1+\eta_{n}\right)}^{z^{\left(t\right)}}\left(1-\alpha \right)\alpha^{z^{\left(t\right)}-1}$ and $b_{1}=\left(1-\alpha \right)\alpha^{z^{\left(t\right)}-1}$. Since $z^{\left(t-1\right)}<z^{\left(t\right)}$, it follows that $a_{2}/b_{2}\geq {\left(1+\eta_{n}\right)}^{z^{\left(t-1\right)}}>{\left(1+\eta_{n}\right)}^{z^{\left(t\right)}}=a_{1}/b_{1}$. We can thus add $z^{\left(t\right)}$ to the sum, and obtain $\left(a_{1}+a_{2}\right)/\left(b_{1}+b_{2}\right)\leq a_{2}/b_{2}$, as desired. Note that this also shows that $\left(a_{1}+a_{2}\right)/\left(b_{1}+b_{2}\right)\geq a_{1}/b_{1}={\left(1+\eta_{n}\right)}^{z^{\left(t\right)}}$, and the induction is complete.

By combining this result with the equivalent argument on the left side, we have shown that we can replace Equation (A10) by

$$\frac{\sum_{x\geq V_{n}^{-}}{\left(1+\eta_{n}\right)}^{-x}\left(1-\alpha \right)\alpha^{x-1}}{\sum_{x\geq V_{n}^{-}}\left(1-\alpha \right)\alpha^{x-1}}\leq \frac{{\overset{ˇ}{M}}_{n}}{M_{n}}=\frac{{\overset{ˇ}{p}}_{n}\left(E_{n}\right)}{p_{\alpha }\left(E_{n}\right)}\leq \frac{\sum_{x>V_{n}^{+}}{\left(1+\eta_{n}\right)}^{x}\left(1-\alpha \right)\alpha^{x-1}}{\sum_{x>V_{n}^{+}}\left(1-\alpha \right)\alpha^{x-1}}$$

or equivalently by

(A12) $${\left(1+\eta_{n}\right)}^{-V_{n}^{-}+1}\frac{\left(1-\alpha \right){\left(1+\eta_{n}\right)}^{-1}}{1-\alpha {\left(1+\eta_{n}\right)}^{-1}}\leq \frac{{\overset{ˇ}{M}}_{n}}{M_{n}}\leq {\left(1+\eta_{n}\right)}^{V_{n}^{+}}\frac{\left(1-\alpha \right)\left(1+\eta_{n}\right)}{1-\alpha (1+\eta_{n})}.$$

In Lemma A1 we have set:

$$\eta_{n}=\epsilon_{n}/\left(1-\epsilon_{n}\right),$$

with

$$\epsilon_{n}:=\sqrt{\frac{\alpha }{\delta n}}\cdot \left(\frac{\max\{1,\frac{1-\alpha }{\alpha }\}}{1-\sqrt{\frac{\alpha }{\delta n}}}\right).$$

On the other hand, by Lemma A2, we have that:

$$V_{n}^{+}\leq \log_{1/\alpha }\left(n\right)+1+\log_{1/\alpha }\left[\frac{1}{(1-\alpha )\delta }\right]$$

and

$$V_{n}^{-}\geq \log_{1/\alpha }\left(n\right)-\log_{1/\alpha }\left[\frac{1}{(1-\alpha )\delta }\right].$$

It follows that both bounds of Equation (A12) converge to 1, at the rate of roughly $\log\left(n\right)/\sqrt{n}$, instead of the parametric rate $1/\sqrt{n}$. Regardless, for any desired η>0, we get that there exists a large enough *n* beyond which, with probability greater than 1−2δ, we satisfy:

$$\frac{1}{1+\eta }\leq \frac{{\overset{ˇ}{M}}_{n}}{M_{n}}\leq 1+\eta .$$

This establishes that ${\overset{ˇ}{M}}_{n}$ learns $M_{n}$, as desired.
